# Cell chirality: its origin and roles in left–right asymmetric development

**DOI:** 10.1098/rstb.2015.0403

**Published:** 2016-12-19

**Authors:** Mikiko Inaki, Jingyang Liu, Kenji Matsuno

**Affiliations:** Department of Biological Sciences, Osaka University, 1-1 Machikaneyama, Toyonaka, Osaka 560-0043, Japan

**Keywords:** cell chirality, left–right asymmetry, f actin, myosin I, cortical inheritance

## Abstract

An item is chiral if it cannot be superimposed on its mirror image. Most biological molecules are chiral. The homochirality of amino acids ensures that proteins are chiral, which is essential for their functions. Chirality also occurs at the whole-cell level, which was first studied mostly in ciliates, single-celled protozoans. Ciliates show chirality in their cortical structures, which is not determined by genetics, but by ‘cortical inheritance’. These studies suggested that molecular chirality directs whole-cell chirality. Intriguingly, chirality in cellular structures and functions is also found in metazoans. In *Drosophila*, intrinsic cell chirality is observed in various left–right (LR) asymmetric tissues, and appears to be responsible for their LR asymmetric morphogenesis. In other invertebrates, such as snails and *Caenorhabditis elegans*, blastomere chirality is responsible for subsequent LR asymmetric development. Various cultured cells of vertebrates also show intrinsic chirality in their cellular behaviours and intracellular structural dynamics. Thus, cell chirality may be a general property of eukaryotic cells. In *Drosophila*, cell chirality drives the LR asymmetric development of individual organs, without establishing the LR axis of the whole embryo. Considering that organ-intrinsic LR asymmetry is also reported in vertebrates, this mechanism may contribute to LR asymmetric development across phyla.

This article is part of the themed issue ‘Provocative questions in left–right asymmetry’.

## 1. Cells are composed of chiral molecules

**Figure 1.**
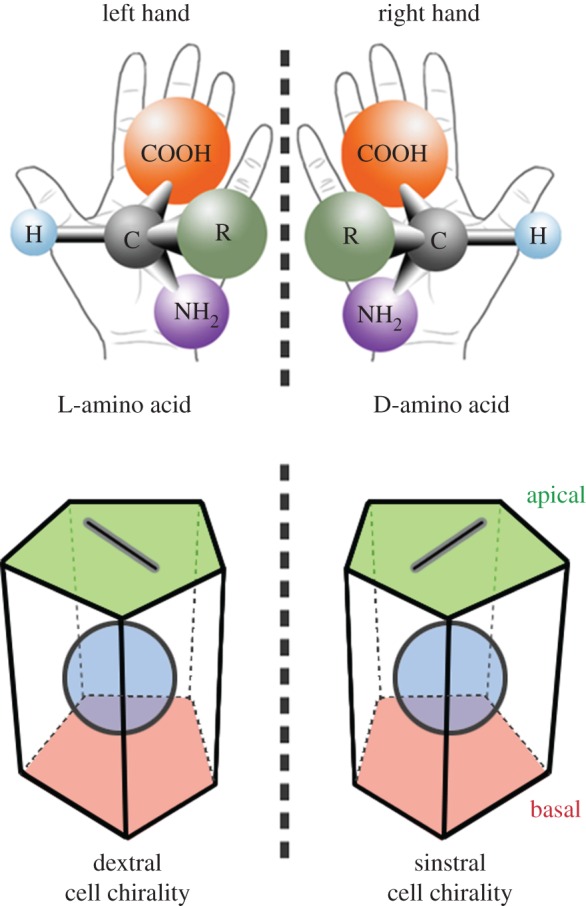
Chirality in hands, molecules and cells. Chirality is a property of an item that cannot be superimposed on its mirror image, as seen in the left and right hands. Most biological molecules, such as amino acids, are chiral. Cells can also be chiral if they have LR asymmetry and apico-basal polarity.

An object or a system is chiral if it cannot be superimposed onto its mirror image. Our left and right hands represent a familiar and convenient example of chirality (figure 1, top). The left hand is a mirror image of the right one, and they cannot be superimposed no matter how the two hands are oriented.

Chirality is a particularly important concept in biology, because cells are mostly composed of chiral molecules. Small chiral molecules such as amino acids and sugars (figure 1, top) are the building blocks of larger molecules, such as proteins and nucleic acids, which are also chiral. A chiral molecule and its mirror image are called enantiomers; one is dextrorotatory (D) and the other is levorotatory (L). Ordinary chemical reactions produce L- and D-molecules in equal amounts, referred to as a racemic mixture. However, related biological molecules have the same chirality; most amino acids are L and most sugars are D. This situation is called homochirality, and the homochirality of biological molecules is a characteristic of all living things. D-amino acids are very rare in cells, although some specific activities of D-amino acids have been identified. For example, in the mammalian brain, D-serine acts as a physiological co-agonist of the *N*-methyl d-aspartate type of glutamate receptor, which is a key excitatory neurotransmitter receptor [1]. However, although the cases in which homochirality is ingeniously used to execute function are uncommon, they demonstrate the importance of chirality in the function of biologically relevant molecules. Interestingly, an enantiomeric excess of L-amino acids was found in the Murchison meteorite, sparking a theory that homochirality has an extraterrestrial origin [2]. In addition, various theories for how a small initial imbalance in enantiomer concentrations could have led to the subsequent production of a single enantiomer have been proposed [3]. For example, the enantioenrichment of biological molecules may have been coupled with the increasing chemical and physical complexity of cells, so the chirality of molecules, including proteins, was intrinsically linked to their functions.

In general, protein functions depend on interactions with other molecules via chiral structures. For a gene to encode a protein with a specific shape, the homochirality of amino acids is required, because L- and D-amino acids will give rise to different three-dimensional protein structures. Thus, the homochirality of amino acids is essential for the basic execution of genetic control. In addition, an enzyme usually has a chiral groove or binding pocket that fits one enantiomer of its substrate but not the other. Thus, the homochirality of biologically active molecules is a critical condition for the molecular functions of organisms.

Chirality may appear less prominent in larger biological structures, although clear exceptions exist. For example, the tail of bacteriophage T4 is a helix with a defined handedness [4], and the structure and motion of prokaryotic flagella are also chiral [5]. However, for most eukaryotic cells, especially metazoan cells, chirality at the single-cell level is not obvious, unlike the marked chirality of the molecules that compose them. Nevertheless, an important role of eukaryotic cell chirality in determining LR asymmetric development in the animal body has recently emerged.

In 1990, Wolpert proposed the ‘F molecule hypothesis’ to explain the development of directional LR asymmetry in the animal body [6]. In this hypothesis, a hypothetical F molecule, which has arms pointing in three dimensions and intrinsic chirality, recognizes the dorsoventral and anteroposterior axes of the embryo and has an activity that arranges it along these two axes. Once the F molecule is placed along the dorsoventral and anteroposterior axes, it defines the LR axis in the embryo by its chirality. More recent evidence indicates that, instead of a chiral molecule like the F molecule directly determining the LR axis in the embryo, chirality at the cellular level dictates the LR asymmetric development in metazoans. However, before exploring the chirality of cells in multicellular eukaryotes and its potential role in complex processes such as LR asymmetric development, we first discuss the chirality found in unicellular eukaryotes, which are a simpler system. In this review, any chirality found at the whole-cell level is referred to as ‘cell chirality’.

## 2. Cell chirality in protozoans, single-celled organisms

**Figure 2.**
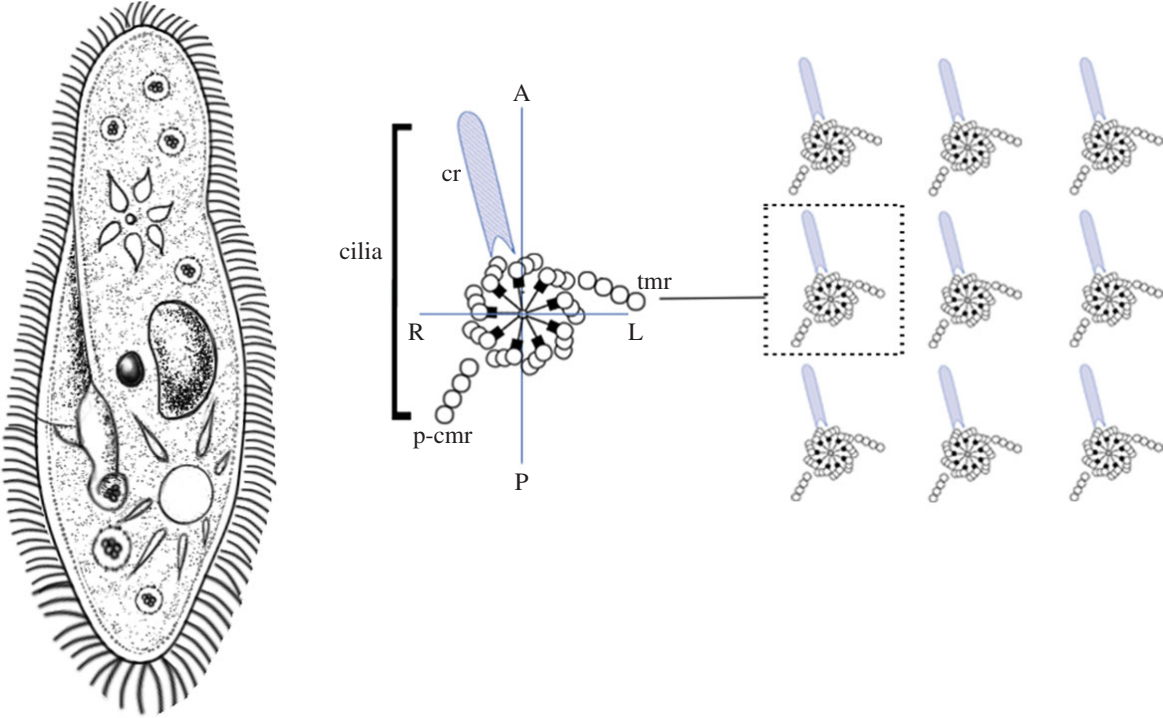
Chirality in ciliates. Right: ciliates show chirality in their global cortical structures, including the ciliary rows, oral apparatus and contractile vacuole. Left: the cortical unit of ciliates, which includes the ciliary rootlet and basal body, is chiral (adapted from [7]). In wild-type ciliates, the ciliary rootlet (cr) extends anteriorly and is positioned to the right relative to the basal body and the cell itself. The transversal microtubule ribbon (tmr) is on the left side of the basal body, and the post-ciliary microtubule ribbon (p-cmr) points posteriorly. Basal bodies are seen from the outside of the cell, and the viewer's right corresponds to the cell's left. Schema is adopted from Beisson [7]. A, anterior; P, posterior; R, right; L, left. (Online version in colour.)

In contrast to multicellular organisms, the protozoans, a diverse group of unicellular eukaryotes, exhibit clear chirality at the cellular level, which has drawn considerable research interest (figure 2, left). The cell chirality in protozoans is an extreme form of cell chirality that may help elucidate the mechanisms of cell chirality formation in metazoans. The ciliates are protozoans that have cilia, which are used for swimming, feeding, sensing and other purposes (figure 2, left). Ciliates exhibit chirality, which is also referred to as ‘handedness’, in their global cortical structures, including the ciliary rows, oral apparatus and contractile vacuole (figure 2, left) [8,9]. The ciliary structure, called the ciliary unit, is positioned in the cell cortex in an asymmetric and polarized (right-handed) manner [7,10]. The ciliary unit is centred over a complex protein structure called the basal body [11,12] (figure 2, right). To examine how polarity forms in ciliates, experimental manipulations were performed to induce atypical ciliary row structures. Stable ciliary phenotypes, including intercalated ciliary rows and mirror-image doublets, can be induced on cells by various techniques, including microsurgery, microbeam laser, thermal shock and chemical shock [13–15]. Notably, such extra sets of ciliary structures can be maintained on the cortex of a clonal cell line for many generations. In addition, in *Tetrahymena*, clones with a global LR asymmetry the reverse of wild-type (left-handed instead of right-handed) were established [9]. Analyses of these left-handed clones revealed that their LR cortical structure is not owing to a genetic change [9]. Collectively, these observations suggest that the existing cortical structural information of a progenitor cell is repeated in its progeny, propagating the cell's global pattern, including its handedness. These analyses also indicated that nuclear genes are not involved in determining handedness [9]. Thus, a pre-existing chiral structure, rather than specific genetic information for cell chirality formation, dictates the cell chirality in the next generation. These phenomena are referred to as ‘cortical inheritance’ or ‘structural memory’, and were a biological mystery for a long time [7,16–18].

Although the molecular mechanisms underlying cortical inheritance are still not completely understood, the cortical unit appears to play an important role in it. The basal body at the base of the cilium and cytoskeletal appendages (called the ciliary rootlet and microtubule ribbon) make up the cortical unit (figure 2, right) [7,11,12]. The ciliary rootlet normally extends in an anterior direction, and on the right side of the basal body and the cell (figure 2, right). The transversal microtubule ribbon is located on the left side of the basal body, and the post-ciliary microtubule ribbon points in a posterior direction [7]. Thus, the cortical unit is chiral (figure 2, right). During cell division, the basal body is duplicated with strict polarity. The newly formed basal body is inserted into the cortex just anterior to its mother, along the longitudinal row of cortical units (figure 2, right) [7,11,12]. Next, cytoskeletal appendages form at the peribasal site, confined within the cortical unit [7]. Thus, in ciliates, properties of the cortical unit itself are sufficient for self-assembly into high-order subcellular structures, such as cytoskeletal organelles and networks [7].

However, nature is even more complex and interesting than one might think. Even in the mirror-image doublets, the mirror-image (enantiomorphic) form of the cortical unit has never been observed [10]. For example, the position of the ciliary rootlet is not the mirror image in the doublet cell [10]. The mirror image oral primordium begins to self-assemble in the normal (right-handed) part of the doublet [10], and then rotates anticlockwise 180° in its plane, resulting in an imperfect mirror image of the oral apparatus [10]. Therefore, in addition to the self-assembly of cortical units, there must be local cues to induce this planar rotation of the cortex. In addition, it was shown that when regions of the cell are placed in abnormal positions relative to one another, the cell intercalates these regions to restore their normal orientations in the membrane by the shortest permissive route [19–22]. These observations led to the proposal that the reversed anteroposterior axis of the oral apparatus in the mirror part of the doublets may be owing to the abnormal juxtapositioning of right and left marginal cortical units [10]. Regardless of the details, cortical inheritance suggests that the LR asymmetric morphology of a cell is dictated by molecular chirality. That is, these observations demonstrate that the chirality of subcellular structures can direct the chirality at the whole-cell level.

## 3. Cell chirality and hindgut laterality in *Drosophila*

**Figure 3.**
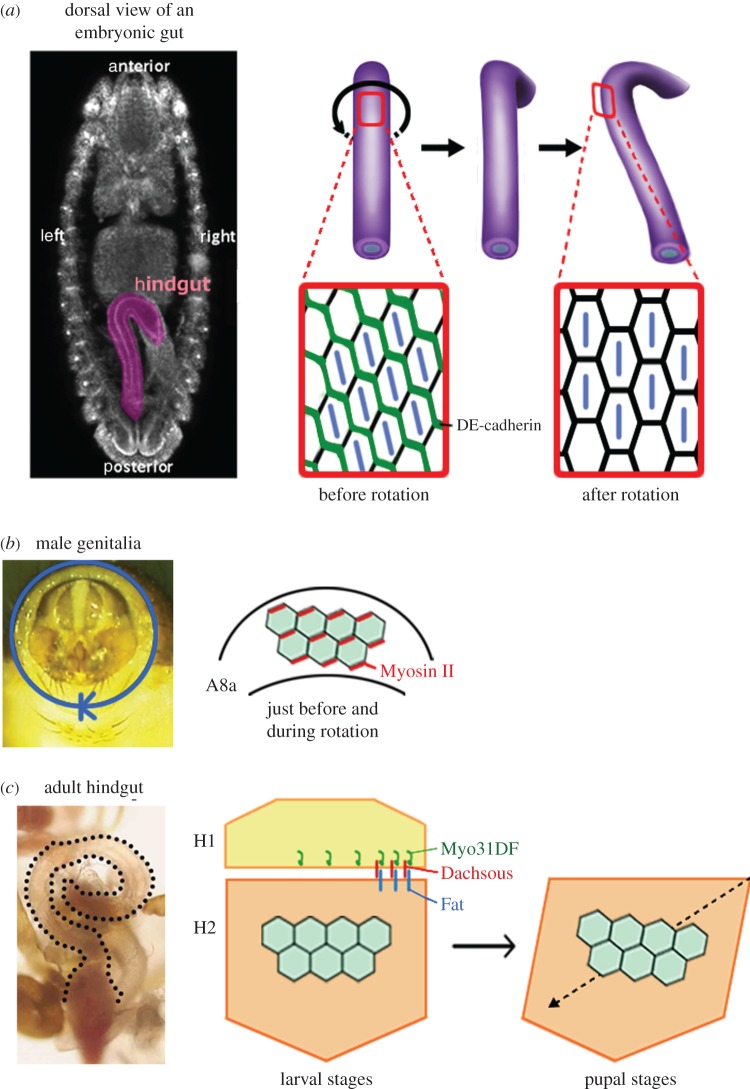
Cell chirality and LR asymmetric morphogenesis in *Drosophila*. (*a*) The *Drosophila* embryonic hindgut shows sinistral looping as the consequence of an LR asymmetric rotation. Before the onset of the rotation, hindgut epithelial cells show chirality with more frequent leftward-tilted cell boundaries than rightward-tilted ones. The chirality disappears when the rotation is completed. Distribution of *D*E-cadherin (green) also shows chirality. (*b*) The *Drosophila* male genitalia undergo a 360° clockwise rotation during the pupal stages. Epithelial cells in the A8a segment of male genitalia show chirality just before and during the LR asymmetric rotation. These cells have more frequent rightward-tilted cell boundaries and a higher expression of Myosin II along the rightward-tilted cell boundaries. Schema is adopted from Sato K *et al.* [23]. (*c*) *Drosophila* adult gut shows LR directional looping. The adult gut develops from larval primordia called the imaginal ring, consisting of H1 and H2 segments. The cell chirality determinant Myo31DF is required only in the H1 segment during larval stages. Cell chirality is observed in the H2 segment only after the H1 segment is eliminated. The handedness determined by Myo31DF in the H1 segment might be conveyed to the H2 segment through atypical cadherins, Dachsous and Fat. Schema is adopted from González-Morales *et al.* [24].

Recent studies revealed that cell chirality is not exclusively found only in protozoans, but also exists in metazoans. Cell chirality in a tissue was first discovered in the *Drosophila* embryonic hindgut, which corresponds to the small and large intestines in mammals (figure 3*a*) [25,26]. The *Drosophila* embryonic hindgut is invaginated from an epithelial monolayer and first forms as a bilaterally symmetric structure. During the late 12 and 13 embryonic stages, the hindgut rotates 90° anticlockwise (as viewed from the posterior) and becomes LR asymmetric with dextral looping (figure 3*a*) [27]. Because the hindgut looping is the first visible sign of LR asymmetry in *Drosophila*, the directional rotation of the hindgut appears to break the LR symmetry. Taniguchi *et al.* [25] discovered that before the directional rotation begins, the apical cell surface of the hindgut epithelial cells shows LR asymmetry (figure 3*a*). These cell surfaces have more leftward-tilted cell boundaries than rightward-tilted ones. Because the hindgut epithelial cells, like other epithelial cells, have apico-basal polarity, their shape is chiral (figure 1, bottom). The cell chirality is evident not only in the overall shape, but also in organelle and protein distributions inside the cells. The centrosomes of hindgut epithelial cells tend to be located in the right-posterior region of the cell, and a cell adhesion molecule *Drosophila* E-cadherin (DE-cadherin) is more abundant along the rightward-tilted cell boundaries than along the leftward-tilted ones at the apical cell surface [25]. This cell chirality diminishes as hindgut rotation progresses and disappears when the rotation is complete (figure 3*a*) [25]. The involvement of the cell chirality in promoting the LR asymmetric rotation of the hindgut was supported by an *in silico* simulation, which showed that the introduction and subsequent dissolution of cell chirality in a model epithelial cell tube is sufficient to recapitulate the directional rotation of the model hindgut [25].

## 4. *Myosin31DF* switches the cell chirality in *Drosophila*

**Figure 4.**
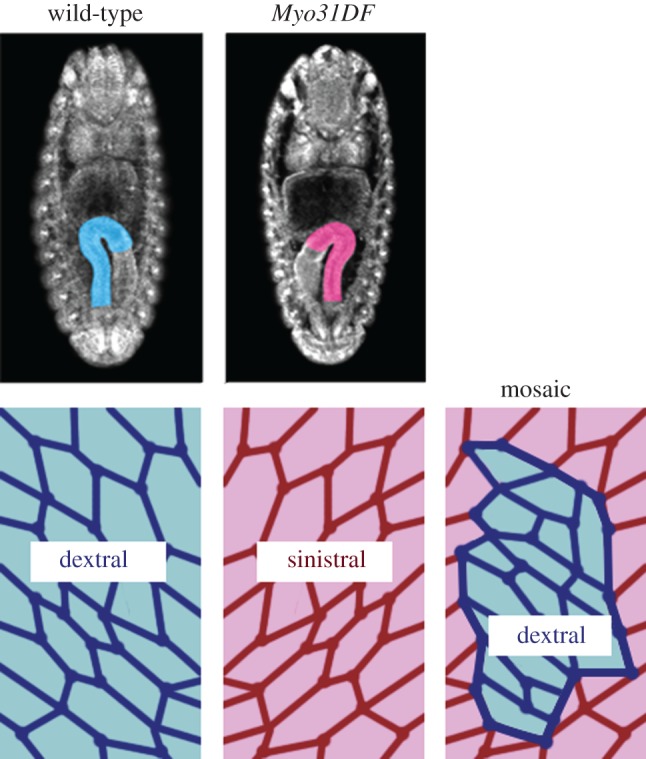
Cell chirality is an intrinsic property of individual cells, and Myo31DF switches the direction of cell chirality. Left: wild-type embryos show rightward looping of the hindgut and dextral cell chirality. Middle: in *Myo31DF* mutant embryos, both the hindgut looping and cell chirality are inverted. Right: when cells expressing wild-type *Myo31DF* are randomly introduced into the *Myo31DF* hindgut, only the cells expressing wild-type *Myo31DF* show the normal dextral chirality, indicating that cell chirality is formed intrinsically in each cell.

Myosin31DF (Myo31DF), an orthologue of mammalian MyosinID, is a key molecule for cell chirality in *Drosophila*. The *Myo31DF* gene was identified in a *Drosophila* screen for gene mutations affecting the LR asymmetry of the embryonic gut [27]. In *Myo31DF* mutants, the embryonic hindgut rotates in the direction opposite to that of wild-type, exhibiting inverted sinistral looping (figure 4) [27]. The cell chirality of the hindgut epithelial cells before the onset of rotation is also inverted in the *Myo31DF* mutants, supporting the notion that the cell chirality prior to rotation is important for the directional rotation in the hindgut (figure 4) [25]. Rescue experiments of *Myo31DF* mutants by wild-type Myo31DF showed that the cell chirality is a cell-autonomous property (figure 4). The inversion phenotypes in both hindgut rotation and cell chirality were rescued by over-expressing wild-type *Myo31DF* in the hindgut epithelial cells [25,28]. When a genetic mosaic was generated by randomly introducing cells expressing wild-type *Myo31DF* in the *Myo31DF* mutant hindgut, wild-type cell chirality was formed only in the cells expressing wild-type *Myo31DF* (figure 4) [28]. These results indicated that cell chirality is intrinsically formed in each cell and that *Myo31DF* functions to switch the cell chirality from the default (*Myo31DF* mutant type) to the wild-type direction (figure 4).

Myo31DF is a member of the unconventional myosin I class; these molecules consist of an N-terminal head domain containing an ATP-binding motif, a neck domain containing two calmodulin-binding IQ motifs, and a short C-terminal tail domain [27,29,30]. A mutant Myo31DF protein lacking the IQ motifs is unable to rescue the *Myo31DF* phenotype [29]. Moreover, mutant Myo31DF proteins lacking the ATP-binding motif, IQ motifs or the tail domain fail to induce LR inversion in the hindgut, unlike wild-type Myo31DF [27]. Myo31DF binds β-catenin and an atypical cadherin, Dachsous, and associates with DE-cadherin through β-catenin [24,31]. Myosin 1d (Myo1d) is a rat orthologue of MyoID. Recently, analyses of a Myo1d knockout rat revealed that Myo1d is required for the formation of planar cell polarity in multiciliated epithelial cells, but not for LR asymmetric organ development [32]. Thus, the roles of MyoID family proteins in LR asymmetric organ development are not evolutionarily conserved in mammals, although their biochemical functions in cell chirality may be widely maintained.

## 5. Cell chirality as a general mechanism of left–right asymmetric development in *Drosophila*

Myo31DF acts as a general LR determinant in *Drosophila* [27,29]. In addition to LR inversion in the embryonic gut, *Myo31DF* mutants exhibit inversion in the looping of the adult gut and testes, and in the rotation of the male genitalia [27,29]. Among these organs, epithelial cells in both the adult gut and the male genitalia show chirality at a point in time related to laterality formation (figure 3*b,c*). *Drosophila* male genitalia undergo a 360° clockwise rotation (as viewed from the posterior) during the late pupal stages [33,34]. This rotation is completed through combined 180° rotations of two segments: the A8 anterior (A8a) and A8 posterior. Sato *et al.* [23] found that epithelial cells in A8a exhibit chirality in their shape and protein distribution. Just prior to and during the directional rotation, these epithelial cells show LR bias, with more frequent rightward-tilted cell boundaries and higher Myosin II expression along the rightward-tilted cell boundaries (figure 3*b*) [23]. The chirality of the A8a cells is reversed in the *Myo31DF* mutant [23]. A computer model demonstrated that the biased cell boundary rearrangement, attributed to the biased expression of Myosin II, is important for the directional rotation of the male genitalia [23].

Another organ in which epithelial cells show chirality is the *Drosophila* adult gut (figure 3*c*). As *Drosophila* undergoes metamorphosis, the adult gut is developed from larval primordia called the imaginal ring. The imaginal ring consists of two segments H1 and H2. Epithelial cells in the H2 segment proliferate during the pupal stages and form the adult gut with dextral looping, whereas the H1 segment is eliminated during the pupal stages [24]. *Myo31DF* activity is required only in H1 during the late larval stages [24]. Interestingly, chirality in the epithelial cell shape is observed only in the H2 segment after the H1 segment is eliminated (figure 3*c*) [24]. González-Morales *et al.* proposed that LR bias generated by Myo31DF in H1 is conveyed to H2 through Dachsous, which physically binds to Myo31DF.

In the *Myo31DF* mutant tissues in which LR asymmetry is the mirror image of wild-type, the cell chirality is also switched from dextral to sinistral (default). Evidence suggests several possible mechanisms for these events. In the epithelium of the *Drosophila* embryonic hindgut, *Myo31DF* is required for the chiral distribution of DE-cadherin [22]. Thus, Myo31DF may act as an LR determinant by regulating the chiral distribution or activation of DE-cadherin. Alternatively, *Myo31DF* may switch the chirality of the structure or function of actin cytoskeleton, given that disrupting the actin cytoskeleton abolishes cell chirality, and that *Myo31DF* is required for the chiral distribution of Myosin II in *Drosophila* [22,34].

## 6. Left–right asymmetry and cell chirality in other invertebrates

Cell chirality–associated phenomena are observed in the blastomeres of various invertebrate species [35]. A spiral cleavage that is conserved in many members of the lophotrochozoan taxa, referred to as Spiralia, often involves chiral blastomeres, especially in the early cleavage stages. In some cases, the chirality of the blastomeres determines the handedness of the embryo.

**Figure 5.**
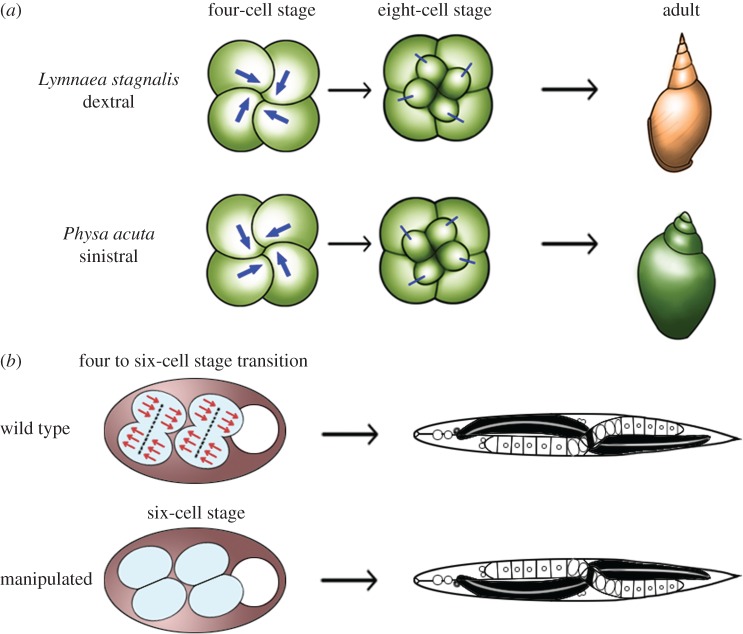
Cell chirality in snails and *C. elegans*. (*a*) Upper: in *Lymnaea*, the blastomere spindles slant clockwise as viewed from the animal pole at the four-cell stage, and blastomeres are rearranged clockwise at the eight-cell stage. Bottom: *Pulmonata* is a snail species with counter clockwise-coiling shells and internal organs that mirror those of *Lymnaea.* In *Pulmonata*, blastomere spindles slant anticlockwise as viewed from the animal pole, resulting in a counter clockwise blastomere rearrangement at the eight-cell stage. In both cases, blastomere chirality determines the shell coiling direction and LR asymmetry of the body. Schema is adopted from Shibazaki *et al.* [39]. (*b*) Top: in *C. elegans*, mitotic spindles are skewed during the transition from four cells to six cells. Bottom: at the six-cell stage, changing the LR-asymmetric arrangement of blastomeres to their mirror-image positions results in situs inversus. Thus, in both snails and *C*. *elegans,* blastomere chirality is completely responsible for the subsequent LR-asymmetric development. Schema is adopted from Wood & Kershaw [40].

Snails, which belong to the Mollusca phylum of the lophotrochozoa, undergo spiral cleavage [36–39]. The directional LR asymmetry of snails is easily observed in the coiling direction of the shell, and the spiral cleavage patterns in snails show a stereotypical handedness (figure 5*a*). In *Lymnaea*, which belongs to the Pulmonata subclass of molluscs, the blastomere spindles slant clockwise (viewed from the animal pole) at the four-cell stage, then the micromeres are rearranged clockwise at the eight-cell stage (figure 5*a*) [39]. Thus, each blastomere at the four-cell stage exhibits cell chirality (figure 5*a*). A formin activity plays a critical role in creating the blastomere chirality in a snail [41], which is reminiscent of a formin-dependent chirality formation seen in mammalian cells, as discussed below [42]. The handedness of the spiral cleavage can be reversed by surgical manipulation at the eight-blastomere stage; these embryos exhibit a mirror-image handedness of their entire body [43]. Therefore, the positioning of blastomeres at the eight-cell stage or earlier determines the handedness of the snail body.

In Pulmonata, mutations affecting the handedness of the shell coiling and internal organs have been found in natural populations [44,45]. In mutants with LR inversion of the shell-coil direction, the early blastomere cleavage pattern is first symmetrical and then becomes a mirror image of the stereotypical cleavage pattern. In Pulmonata evolution, species occasionally emerged with anticlockwise-coiling shells and internal organs that were mirror images of those in the dextrally coiling snails [39]. The spiral blastomere cleavage pattern in these sinistrally coiling species is also the mirror image of the pattern seen in the dextrally coiling snails (figure 5*a*) [39]. These studies showed that the handedness of the spiral cleavage is correlated with the direction of shell coiling and of the internal organs [39]. Interestingly, the first cleavage in *Xenopus* is accompanied by a slight anticlockwise torsion of the two blastomeres [46]. A chemical treatment can dramatically increase this cortical anticlockwise torsion, and pharmacological analyses suggested that the torsion requires F-actin [46]. Thus, the cortex of an egg undergoing radial cleavage has intrinsic chirality, supporting the idea that cell chirality is a common property in metazoans.

*Caenorhabditis elegans* (*C*. *elegans*) is an Ecdysozoan model animal that has stereotypic LR asymmetry of the body [40]. As in snails, the first sign of LR asymmetry in *C*. *elegans* is an anterior–posterior skewing of the transverse mitotic spindles with predetermined laterality, at the four-cell stage [47] (figure 5*b*). At the eight-cell stage, the embryo midline tilts rightward from the anterior–posterior axis; this positioning is induced by LR-asymmetric blastomere protrusion and migration [48]. These events involve differentially regulated cortical contractility in the sister blastomeres that are bilateral counterparts [48]. Changing the LR-asymmetric blastomere configuration at the six-cell stage to their mirror-image positions causes situs inversus [40] (figure 5*b*). Thus, as with snails, the relative LR-asymmetric blastomere positioning is completely responsible for the subsequent LR-asymmetric body development in *C*. *elegans* [40,43]. That is, intercellular interactions responsible for the subsequent LR-asymmetric development depend on the LR-asymmetric blastomere configuration in the early cleavage stages. In summary, blastomere chirality is a common mechanism driving LR asymmetric development in various invertebrates. Although the molecular mechanisms underlying blastomere chirality formation are not well understood at present, it may have common features with other cases of cell chirality formation, such as the involvement of formin and actin, as discussed below.

## 7. Cell chirality in vertebrate cultured cells

Cell chirality was recently observed in various vertebrate cultured cells. For example, murine myoblast C2C12 cells, human umbilical vein endothelial cells (hUVECs) and vascular mesenchymal cells (VMCs) show a chirally polarized cell shape when plated on a micropattern [49,50]. Whether the handedness is dextral or sinistral depends on the cell line [49]. Chirality in the nuclear shape and the involvement of E-cadherin in transmitting a chiral bias to neighbouring cells were shown using Madin–Darby canine kidney epithelial cells [51,52].

**Figure 6.**
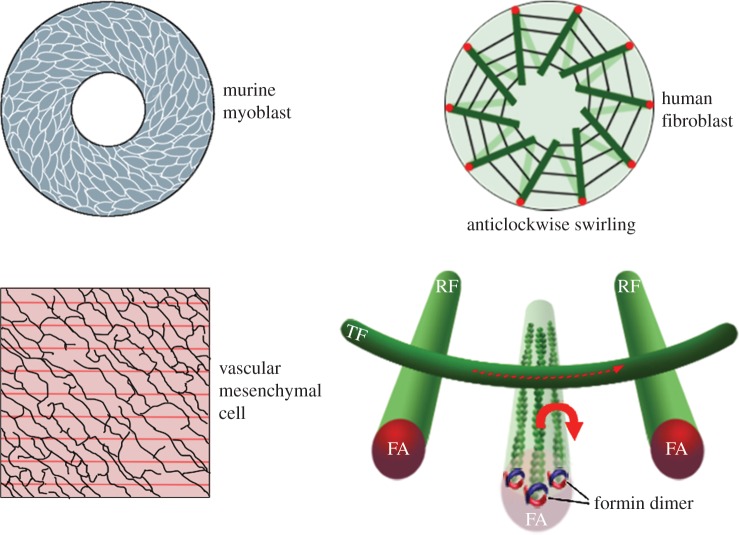
Chiral shape and swirling of cultured mammalian cells. Left: cultured murine myoblasts (top) and vascular mesenchymal cells (bottom) demonstrate intrinsic chirality when plated on a substrate with a ring or stripe micropattern. Schemas are adopted from Wan *et al.* [49] and Chen *et al.* [50]. Right: fibroblasts from human foreskin seeded on a micropattern show anticlockwise chiral swirling. Radial actin fibres initially situated in a radial pattern eventually start to tilt rightward (top). The clockwise rotation of actin filaments in radial fibres generated by formin may cause the rightward tilting (bottom). Schemas are adopted from Tee *et al.* [42].

Cell chirality is also observed in the dynamics of cultured cells. Human promyelocytic leukaemia (HL60) cells, which are neutrophil-like cells, show a leftward-biased migration in the absence of spatial cues [53]. Genetic and pharmacological analyses revealed that microtubules are involved in this process [53]. Fibroblasts from human foreskin seeded on a micropattern and cultured zebrafish melanophores show chiral swirling [42,54]. In these processes, the actin cytoskeleton is important, but microtubules are not [42,54]. Tee *et al.* studied the detailed molecular mechanisms underlying this fibroblast swirling. They found that fibroblasts seeded on a circular micropattern develop two types of actin fibres, radial and transverse, and that the radial fibres eventually start to tilt unidirectionally, generating the chiral swirling (figure 6) [42]. This process was found to require the radial growth of the radial fibres, which depends on actin's polymerization by formin [42]. Formin appears to give a unidirectional rotation to actin filaments, which results in a rightward tilting of radial fibres when triggered by a slight imbalance in transverse fibres (figure 6). Interestingly, α-actinin-1, an actin filament bundling protein, appears to act as a chirality switch in this system. Over-expressing α-actinin-1 changes the direction of the chiral swirling from anticlockwise to clockwise [42].

## 8. Implications

Directional LR asymmetry of the body structure is broadly observed in ecdysozoans, lophotrochozoans and deuterostomes. In addition, cell chirality is observed in these three groups of animals. Thus, it is possible that the mechanisms by which chiral morphology develops, including cell chirality, can be traced back to the ancestral bilateralia. In the cases of cell chirality observed so far, the actin cytoskeleton appears to play a profound role. In particular, formin, which drives the unidirectional rotation of F-actin, is indispensable for the formation of cell chirality in snail, frog and mammalian cells [41,42]. Thus, chirality in the structure or function of actin cytoskeleton may be an important determinant of cell chirality.

**Figure 7.**
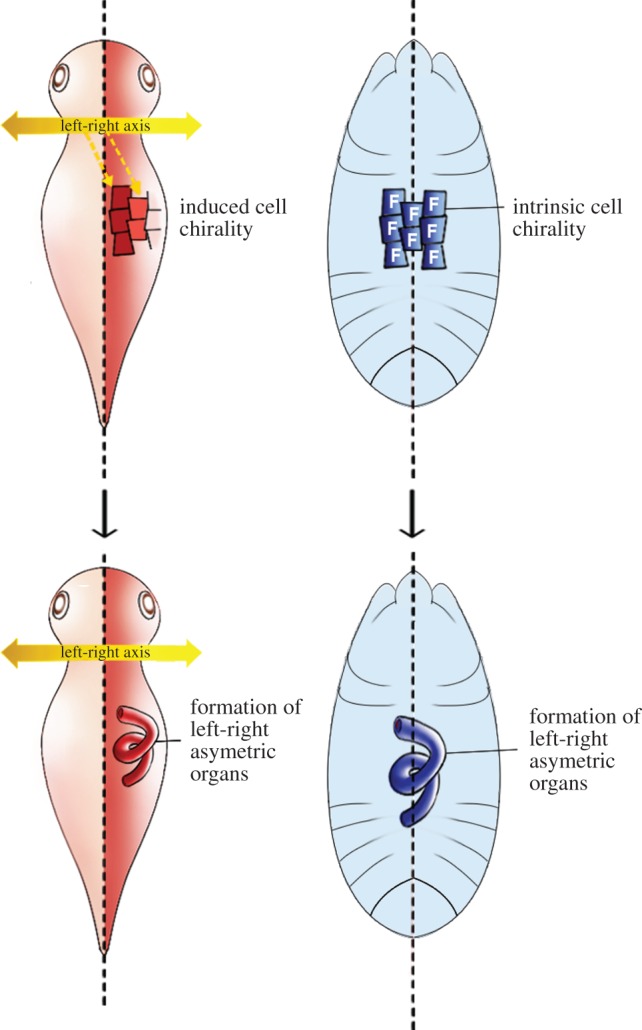
The ‘F cell’ concept and LR asymmetric development in the absence of an LR axis. Left: in vertebrates, LR morphogenesis occurs according to an established body LR axis. Right: in *Drosophila*, chiral cells may behave like an F cell, which is analogous to the F molecule—a hypothetical LR determinant—at the cellular level and drive LR asymmetric development in individual organs, without establishing an LR axis of the whole embryo.

During animal development, most cells differentiate and exhibit functions at specific parts of the embryo, which are determined by positional information based on the dorsoventral and anteroposterior axes. Given that some of these cells have intrinsic cell chirality and are positioned in a specific part of the embryo, these cells can define the LR polarity, leading to LR asymmetric development, as found in *Drosophila*. In this case, chiral cells behave like an F cell, which is equivalent to the F molecule at the cellular level, and drive LR asymmetric development individually in each organ, without establishing an LR axis of the whole embryo (figure 7). This scenario is supported by the absence of any observed LR-asymmetric gene expression in *Drosophila*. Therefore, cell chirality may serve as a mechanism for inducing organ-intrinsic LR asymmetry in the absence of an established LR axis [23–25].

In vertebrates, later LR morphogenesis (such as the position and morphology of internal organs) is influenced by an established body LR axis, which is achieved by Nodal signalling [55,56]. In addition, Nodal-independent LR-asymmetric organ morphogenesis was recently reported in a vertebrate. In a zebrafish mutant defective for the Nodal-related gene *Southpaw*, the left-side–specific gene expression is abolished as expected; however, these mutants still show a dextral looping structure in the heart [57]. Moreover, explanted linear heart tubes from chicks or fish develop dextral looping in culture [57–59], indicating that this morphogenesis is independent of the LR body axis; that is, that organ-intrinsic mechanisms of LR-asymmetric development like those found in *Drosophila* may also occur in vertebrates. Given that many types of cells from various organs and organisms show cell chirality, mechanisms driven by cell chirality might be a common platform for the development of organ-intrinsic LR asymmetry.
